# Department of Health, Buffalo, N. Y.

**Published:** 1910-10

**Authors:** F. E. Fronczak

**Affiliations:** Health Commissioner


### CORRESPONDENCE

DEPARTMENT OF HEALTH, BUFFALO, N. Y.
*Division of Contagious Diseases.*

*Editor Buffalo Medical Journal*:

Sir—The Department of Health will hereafter require two *negative* cultures from a diphtheria patient before removal of quarantine, the first culture to be taken by the attending physician and the second by an inspector from this department.

In other words, the attending physician will, as heretofore, make as many cultures as may be necessary until the result is negative, after which an inspector will make the final culture.

Whenever, in the judgment of this department, it is necessary, cultures will also be taken of other members of the family or household of the patient.

This step has been determined upon in order to eliminate possible carriers, and also with a view of minimising the dangers from diphtheria during the coming season.

F. E. FRONCZAK, M. D.,
August 15, 1910. Health Commissioner.

